# Sarcopenia, long‐term conditions, and multimorbidity: findings from UK Biobank participants

**DOI:** 10.1002/jcsm.12503

**Published:** 2019-12-30

**Authors:** Richard M. Dodds, Antoneta Granic, Sian M. Robinson, Avan A. Sayer

**Affiliations:** ^1^ AGE Research Group, NIHR Newcastle Biomedical Research Centre Newcastle University and Newcastle upon Tyne NHS Foundation Trust Newcastle upon Tyne UK; ^2^ Newcastle University Institute for Ageing Newcastle upon Tyne UK

**Keywords:** Sarcopenia, Grip strength, Long‐term conditions, Multimorbidity, Mid‐life, Later life

## Abstract

**Background:**

Sarcopenia, the loss of muscle strength and mass, predicts adverse outcomes and becomes common with age. There is recognition that sarcopenia may occur at younger ages in those with long‐term conditions (LTCs) as well as those with multimorbidity (the presence of two or more LTCs), but their relationships have been little explored. Our aims were to describe the prevalence of sarcopenia in UK Biobank, a large sample of men and women aged 40–70 years, and to explore relationships with different categories of LTCs and multimorbidity.

**Methods:**

We used data from 499 046 participants in the baseline of UK Biobank. Our main outcome was probable sarcopenia based on maximum grip strength below sex‐specific cut‐points. Participants' LTCs were recorded during an interview and categorized against a hierarchy. We used logistic regression to examine the independent associations between each category of LTCs and probable sarcopenia, including adjustment for age, sex, and body mass index. We also examined the association with multimorbidity.

**Results:**

Probable sarcopenia had an overall prevalence of 5.3% and increased with age. The categories with the strongest associations with probable sarcopenia were musculoskeletal/trauma [OR 2.17 (95% CI: 2.11, 2.23)], endocrine/diabetes [OR 1.49 (95% CI: 1.45, 1.55)], and neurological/psychiatric [OR 1.39 (95% CI: 1.34, 1.43)] LTCs. Almost half of the sample (44.5%) had multimorbidity, and they were at nearly twice the odds of probable sarcopenia [OR 1.96 (95% CI: 1.91, 2.02)] compared with those without.

**Conclusions:**

We have shown an overall prevalence of 5.3% of probable sarcopenia at ages 40–70 in UK Biobank. The risk of probable sarcopenia was higher in those with some categories of LTCs, suggesting that these groups may stand to benefit from assessment of sarcopenia, during mid‐life as well as old age.

## Introduction

Sarcopenia, the accelerated loss of muscle strength and mass, is important as it predicts a range of adverse outcomes^1^, ^2^ and it is amenable to interventions including resistance exercise training and nutritional supplementation.^3^ The recent European Working Group on Sarcopenia in Older People 2 (EWGSOP2) consensus definition enables the clinical identification of sarcopenia in several respects.^4^ Firstly, it recommends cut‐points for low muscle strength and mass. Secondly, it introduces the concept of probable sarcopenia: the presence of low strength based on poor performance in grip strength or chair rise tests, as a basis on which to begin intervention when assessment of muscle mass is not available. Finally, it highlights that sarcopenia is primarily an age‐related condition but is also thought to occur at younger ages secondary to the presence of long‐term conditions.

The links with long‐term conditions mean that sarcopenia is of growing interest to a wide range of clinical specialties.^5^ Studies highlight an increased prevalence of sarcopenia in those with chronic obstructive pulmonary disease,^6^ chronic heart failure,^7^ and chronic liver disease.^8^ Interactions with skeletal muscle may also be important in the pathogenesis of long‐term conditions, for example, osteoporosis^9^ and heart failure.^10^ Long‐term conditions also do not typically occur in isolation. Multimorbidity, commonly defined as the presence of two or more long‐term conditions, is the norm among users of health care and those aged 65 and above.^11^ It is associated with a range of adverse outcomes including all‐cause mortality, increased health care costs, reduced quality of life, depression, and functional limitation.^12^, ^13^, ^14^ There has been comparatively little research into the overall relationship between sarcopenia and multimorbidity. Previous work includes the finding that the number of long‐term conditions present in an individual is inversely related to their grip strength^15^, ^16^, ^17^, ^18^ and muscle mass.^19^ More recently, it has been recognized that some categories of long‐term conditions are more strongly associated with impairments in physical function than others.^20^ An understanding of which categories of long‐term conditions pose the greatest risk of sarcopenia would be helpful as it could focus the assessment of sarcopenia to those most likely to benefit from interventions.

The aims of the present study were therefore (i) to describe the prevalence of sarcopenia in a large sample of adults including those in middle age and (ii) to investigate the relationships between probable sarcopenia and different categories of long‐term conditions, in terms of individual categories and multimorbidity (the number of categories present).

## Methods

UK Biobank is a large prospective epidemiological study designed to investigate the roles of genetic, lifestyle, and environmental factors in health and disease in mid‐life to later life.^21^ In summary, 502 536 participants aged 37–73 were recruited and seen for baseline assessment at 22 centres in England, Wales, and Scotland between 2006 and 2010. Ethical approval for UK Biobank was obtained from the North West Multi‐Centre Research Ethics Committee.

### Assessment of probable and confirmed sarcopenia

Grip strength was measured at baseline in UK Biobank by a trained research nurse using a Jamar handheld dynamometer. One measurement was taken from both hands with the participant in the seated position with their forearms on armrests. We used the maximum of the available values.^22^ We used the cut‐points for weak grip strength (indicating probable sarcopenia) as recommended in the EWGSOP2 definition of <27 kg in men and <16 kg in women.^4^, ^23^ We considered those unable to perform grip strength measurement in either hand due to health reasons (arthritis, previous stroke, or other health problems, meaning that they were unable to perform the test) as having weak grip strength for the purpose of analyses.

Appendicular fat‐free mass was measured using bioimpedance analysis with a Tanita BC‐418MA body composition analyser.^24^ Those with a pacemaker, those unable to stand, those unwilling to remove their shoes, or women who reported that there was any possibility they might be pregnant were excluded. We used data from UK Biobank participants (*n* = 4350) who underwent a dual‐energy x‐ray absorptiometry in a later assessment to produce an equation to estimate appendicular lean mass (ALM) from the appendicular fat‐free mass values: ALM (kg) = (0.958 × [Appendicular fat‐free mass (kg)]) − (0.166 × *G*) − 0.308, with *G* taking value 0 if female and 1 if male. For full details, please see the Supplementary Methods section in the supporting information.

Standing height was measured using a Seca 202 height measure, and we expressed ALM relative to height squared. We used the cut‐points as recommended in the EWGSOP2 definition of <7 kg/m^2^ in men and 6 kg/m^2^ in women.^4^

We considered all participants with weak grip strength to have at least probable sarcopenia, including those who had not completed ALM measurement. We considered those with weak grip strength and low ALM to have confirmed sarcopenia. UK Biobank did not include measurement of physical performance although participants were asked to describe their own walking pace as unable to walk, slow, steady average, or brisk. We therefore considered participants with confirmed sarcopenia and who reported that they were unable to walk or walked at a slow pace to have low physical performance^25^ to have severe sarcopenia.

### Assessment of long‐term conditions, multimorbidity, and other characteristics

Participants were asked if they had ever been told by a doctor that they had one or more of the following illnesses: heart attack, angina, stroke, high blood pressure, blood clot in leg, blood clot in lung, emphysema/chronic bronchitis, asthma, or diabetes. Those who reported any of those conditions, or of having a history of cancer or any other serious illness or disability, were then asked to complete an interview in which a research nurse recorded details of all long‐term conditions against a hierarchical tree of over 450 conditions similar to that used in the ICD‐10 classification.^26^ We summarized the responses as binary variables for the presence or absence of one or more conditions in each top‐level category, with a separate variable for those reporting a history of one or more cancer(s). We categorize the number of categories each participant had into none, one, two, and three or more, with the latter two considered to represent multimorbidity.

Weight was measured at the same time as the bioimpedance measurement. We expressed body mass index (BMI) (weight/height^2^) both as a continuous variable and using standard cut‐points. Participants were asked if they had falls in the last year, classified as none, one, or two or more.

### Statistical analyses

We excluded the small number of participants aged under 40 or over 70 and those with missing data for grip strength, height, weight, or long‐term conditions (see Supporting Information, *Figure S1*). For the main analyses, we classified participants into either no sarcopenia (normal grip strength) or probable sarcopenia (weak grip strength). We produced descriptive statistics for each group and used logistic regression to explore the associations between each category of long‐term condition and probable sarcopenia, including adjustment for age, sex, BMI category, and other categories of long‐term conditions.

In further analyses, we produced descriptive statistics for those in the main analyses with probable sarcopenia and measurement of ALM, divided into those with ALM‐for‐height above and below the EWGSOP2 cut‐points. We performed all analyses using Stata version 14.0.^27^

## Results

### The prevalence and characteristics of participants with probable sarcopenia

A total of 499 046 participants had information available on grip strength, BMI, and the presence of long‐term conditions (for more details, see Supporting Information, *Figure S1*). Probable sarcopenia had an overall prevalence of 5.3% and was associated with fall(s) in the last year and reporting being unable to walk or walking at a slow pace as shown in *Table* 1. Probable sarcopenia became more common with age: 2.5%, 4.5%, and 7.6% in the 40–49, 50–59, and 60–70 age groups, respectively.

**Table 1:** Characteristics of sample by probable sarcopenia status

Characteristic [*N* = 499 046 and all values *n* (%) unless shown]	No sarcopenia *n* = 472 375 (94.7%)		Probable sarcopenia *n* = 26 671 (5.3%)	
Age [mean (SD)]	56.3	(8.1)	59.9	(7.2)
Age category				
40–49	114 083	(24.2)	2948	(11.1)
50–59	158 618	(33.6)	7422	(27.8)
60–70	199 674	(42.3)	16 301	(61.1)
Female gender	255 332	(54.1)	16 407	(61.5)
Maximum grip (kg) [mean (SD)]				
Women	25.9	(5.6)	11.8	(3)
Men	42.6	(8.1)	22.1	(4.3)
BMI (kg/m^2^) [mean (SD)]	27.4	(4.8)	28.1	(5.4)
BMI category				
<18.5	2341	(0.5)	280	(1)
18.5 ≤ BMI < 25	154 638	(32.7)	7667	(28.7)
25 ≤ BMI < 30	201 391	(42.6)	10 582	(39.7)
BMI ≥ 30	114 005	(24.1)	8142	(30.5)
Conditions(s) by category				
Cardiovascular	169 664	(35.9)	13 370	(50.1)
Respiratory/ENT	82 992	(17.6)	6122	(23)
Gastrointestinal	73 916	(15.6)	6119	(22.9)
Renal/urology	21 651	(4.6)	1711	(6.4)
Endocrine/diabetes	48 457	(10.3)	4985	(18.7)
Neuro/psych.	64 888	(13.7)	5351	(20.1)
Musculoskeletal/trauma	97 979	(20.7)	11 139	(41.8)
Haematology/dermatology	25 635	(5.4)	1918	(7.2)
Gynaecology/breast	25 271	(5.3)	1824	(6.8)
Immunological/systemic	39 970	(8.5)	2564	(9.6)
Infections	8767	(1.9)	654	(2.5)
Eye	17 110	(3.6)	1833	(6.9)
History of cancer	38 594	(8.2)	2905	(10.9)
Number of categories affected				
0	117 311	(24.8)	3077	(11.5)
1	150 077	(31.8)	6307	(23.6)
2	108 853	(23)	6824	(25.6)
3+	96 134	(20.4)	10 463	(39.2)
ALM/height^2^ (kg/m^2^) [mean (SD)] (*n* = 491 551)				
Women	7.1	(0.8)	7.2	(1)
Men	8.8	(1.1)	8.7	(1.3)
Falls in last year (*n* = 496 880)				
0	381 162	(81)	17 639	(67.1)
1	61 103	(13)	4415	(16.8)
2+	28 321	(6)	4240	(16.1)
Self‐reported walk speed (*n* = 496 845)				
Unable to walk	1166	(0.2)	401	(1.5)
Slow pace	33 261	(7.1)	6966	(26.5)
Steady average pace	248 064	(52.7)	13 550	(51.6)
Brisk pace	188 070	(40)	5367	(20.4)

ALM, appendicular lean mass; BMI, body mass index; ENT, ear nose and throat; SD, standard deviation.

**Figure 1:**
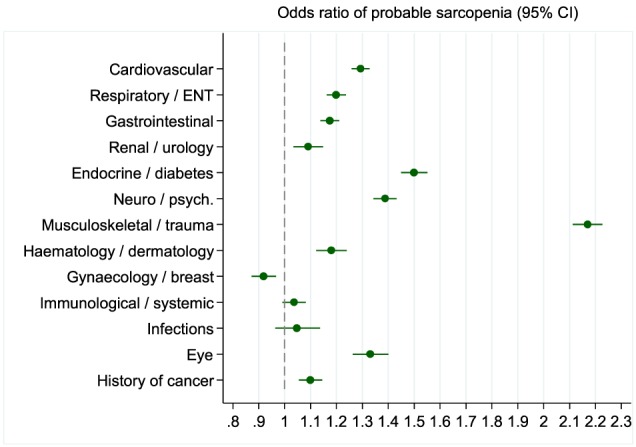
Independent associations between probable sarcopenia and each category of chronic conditions. Findings from logistic regression model showing independent associations between having one or more chronic conditions in each category and probable sarcopenia. Other variables in model (not shown): age (as linear term), sex, and body mass index category. *N* = 499 046. ENT, ear nose and throat.

### The relationship between probable sarcopenia, long‐term conditions, and multimorbidity

Those with probable sarcopenia were more likely to have each category of long‐term conditions, as shown in *Table* 1. For example, 50.1% of those with probable sarcopenia had one or more cardiovascular conditions compared with 35.9% of those without (*P* < 0.001). Most categories of long‐term conditions continued to have associations with probable sarcopenia, following adjustment for the other categories, BMI, and gender as shown in *Figure* 1. The strongest independent associations were seen for musculoskeletal/trauma conditions [OR 2.17 (95% CI: 2.11, 2.23)], endocrine/diabetes [OR 1.49 (95% CI: 1.45, 1.55)], neurological/psychiatric [OR 1.39 (95% CI: 1.34, 1.43)], eye conditions [OR 1.33 (95% CI: 1.26, 1.4)], and cardiovascular conditions [OR 1.29 (95% CI: 1.26, 1.33)].

**Figure 2:**
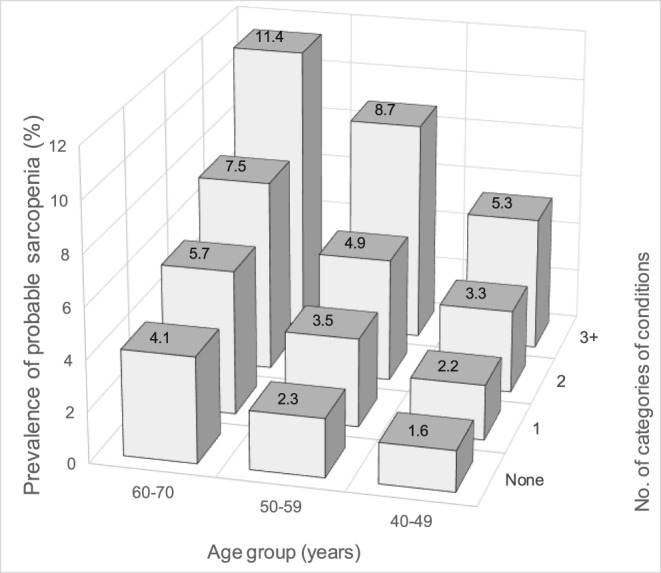
Prevalence of probable sarcopenia by age and number of categories of conditions. The prevalence of probable sarcopenia is shown for each combination of age group and number of categories of chronic conditions. Men and women combined, *N* = 499 046.

Almost half of the sample (44.5%) had multimorbidity (two or more categories of long‐term conditions). This was more common in those with probable sarcopenia (64.8%) than those without (43.4%). The increase in the prevalence of probable sarcopenia with both age and number of categories of long‐term conditions is shown in *Figure* 2. Those with multimorbidity had almost twice the odds of probable sarcopenia compared with those without [OR 1.96 (95% CI: 1.91, 2.02)], after adjustment for age, sex, and BMI category.

### The prevalence and characteristics of participants with confirmed sarcopenia

Of those with probable sarcopenia, 770 (2.9%) did not have measurement of ALM. Compared with those with probable sarcopenia who had ALM measurement, this group was more likely to be male, to have weaker grip strength, to be obese, to have three or more systems affected by long‐term conditions, to report two or more falls in the last year, and to report walking at a slow pace (see Supporting Information, *Table S1*). Among the 25 091 participants with probable sarcopenia and who had a measurement of ALM, 1670 (6.4%) had confirmed sarcopenia (low ALM‐for‐height), a prevalence of 0.3% among the entire sample.

The characteristics of participants with confirmed sarcopenia are shown in Supporting Information, *Table S2*. When compared with those with weak grip and normal ALM‐for‐height, many of their characteristics were similar. However, a key difference seen was in BMI, such that underweight was much more common, with a mean (standard deviation) BMI: 19.6 (2.0) kg/m^2^ among those with confirmed sarcopenia compared with 28.3 (5.3) kg/m^2^ among those without. Of those with confirmed sarcopenia, 404 (24.5%) reported being unable to walk or walking at a slow pace, and we therefore considered them to have severe sarcopenia. This was in fact a smaller proportion than that seen in the weak grip and normal ALM‐for‐height group: 27.6% (*P* < 0.001).

## Discussion

### Summary of findings

We found an overall prevalence of probable sarcopenia of 5.3% among UK Biobank participants aged 40–70 years at the time of assessment. We saw independent associations of probable sarcopenia with most categories of long‐term conditions, particularly the musculoskeletal/trauma, endocrine/diabetes, neurological/psychiatric, eye, and cardiovascular categories. The prevalence of probable sarcopenia increased with both age and the number of categories of long‐term conditions, as illustrated by the finding of a similar prevalence in those aged 40–49 with three or more categories of long‐term conditions to those aged 60–70 with only one. Participants with multimorbidity (two or more categories of long‐term conditions) were at almost twice the odds of having probable sarcopenia compared with those without. Finally, we saw a low prevalence of confirmed sarcopenia of 0.3%. The main difference between those with confirmed sarcopenia and those with weak grip and normal muscle mass was that those with confirmed sarcopenia had lower average BMI.

### Comparison with existing studies

The concept of probable sarcopenia is a recent one, and we are not aware of other studies that have assessed its prevalence in community settings. We are also not aware of studies that have examined the prevalence of sarcopenia at younger ages, with a recent systematic review reporting a minimum age of 55 years across 109 prevalence studies.^28^ Finally, we are not aware of studies that have specifically examined associations between long‐term conditions and sarcopenia, although two closely related areas of investigation are the associations of multimorbidity with grip strength as a continuous variable and with other measures of physical function, as described in the succeeding texts.

Several studies have shown inverse relationships between hand grip strength and the total number of long‐term conditions in older individuals.^15^, ^16^, ^17^, ^18^, ^29^ The present analyses are consistent with this evidence, in which we have shown this relationship using the EWGSOP2 cut‐points for weak grip strength. However, a striking finding is that the association is also present in younger adults. Our analyses have highlighted considerable heterogeneity in the relationship between long‐term conditions and weak grip strength, with some categories of long‐term conditions having stronger independent associations with probable sarcopenia than others. This is in keeping with findings from the development and validation of a weighted index for the relationship between different long‐term conditions and self‐reported physical function,^20^, ^30^ which included long‐term conditions in the same top five categories as we found for probable sarcopenia, along with respiratory disease and a history of non‐skin cancer. Two of these categories, cardiovascular and neuropsychiatric conditions, have also been found to predict declines in walking speed and activities of daily living.^31^

The recent definition of probable sarcopenia is intended to allow recognition and management of sarcopenia in a clinical setting, where it is acknowledged that measurement of muscle mass may not be possible.^32^ Indeed, a proportion of the current sample did not undergo bioimpedance analysis, and this group appeared to be in worse health than those who did. In clinical practice, it may therefore be relevant to note when this test is not completed as it may identify a group at risk of worse outcomes. Among those with probable sarcopenia who completed bioimpedance analysis, the main difference we observed between those with confirmed sarcopenia and those with weak grip but normal ALM‐for‐height was that the former group had lower BMI. We previously noted this in a study of 85 year olds.^33^

### Interpretation of findings

Probable sarcopenia is an example of impaired physical function that is thought to have complex relationships with individual long‐term conditions and multimorbidity, including bidirectional effects.^34^ The associations we have demonstrated may be due to confounding, for example, by lifestyle risk factors such as lower physical activity. However, there are also several possibilities by which the associations may be causal. Firstly, other studies highlight that adult grip strength and some long‐term conditions have common genetic factors.^35^, ^36^ Secondly, arthritis such as at the wrist joint may lead to reduced grip strength. Thirdly, it is likely that some long‐term conditions have deleterious effects on skeletal muscle tissue. For example, patients with diabetes have been shown to have higher levels of collagens surrounding muscle fibres^37^ that may impair force transmission and also lead to reduced expression of mitochondrial genes.^38^ Alternatively, medications used to manage some long‐term conditions, such as oral steroids, may cause loss of muscle mass as a side effect. An improved understanding of the biological mechanisms underlying these possibilities is needed if we are to prevent and treat probable sarcopenia in the setting of multimorbidity.

### Methodological considerations

Whilst UK Biobank is recognized to have a degree of healthy responder bias^39^ and hence, the prevalence estimates in this study are likely to be lower than the values in the general population, the large sample size and mid‐life focus of UK Biobank allowed us to investigate the independent associations of each category of long‐term conditions, including at younger ages than in previous sarcopenia studies. UK Biobank did not include the chair stand test, proposed by EWGSOP2 as an alternative to grip strength for identifying probable sarcopenia, and it also did not include tests such as walking speed to allow us to identify those with severe sarcopenia (although we were able to use reported walking speed as a proxy).

In order to make the findings generalizable in other settings, we used categories of long‐term conditions as our exposures, although an alternative would have been to examine associations with individual conditions. Finally, an area for future work would be to explore if particular clusters of categories have stronger associations with sarcopenia than suggested by their individual associations.^40^

## Conclusions

We have shown an overall prevalence of probable sarcopenia at ages 40–70 in UK Biobank of 5.3%, with the risk increasing with age. We have also shown that probable sarcopenia is associated with specific categories of long‐term conditions as well as multimorbidity at ages 40 and above. The strength of association between different categories of long‐term conditions and probable sarcopenia varied, with musculoskeletal/trauma, endocrine/diabetes, neurological/psychiatric, eye, and cardiovascular conditions appearing to confer the greatest risk. These findings are important as they suggest that particular groups could be targeted for interventions in mid‐life aimed at the prevention and treatment of sarcopenia.

## Funding

A.A.S. is Director of the NIHR Newcastle Biomedical Research Centre. The research was supported by the National Institute for Health Research (NIHR) Newcastle Biomedical Research Centre based at Newcastle upon Tyne Hospitals NHS Foundation Trust and Newcastle University. The views expressed are those of the author(s) and not necessarily those of the NHS, the NIHR, or the Department of Health.

## Conflict of interest

None declared.

## Ethical approval

Ethical approval for UK Biobank was obtained from the North West Multi‐Centre Research Ethics Committee. The authors certify that they comply with the ethical guidelines for publishing in the *Journal of Cachexia, Sarcopenia and Muscle*: update 2017.^41^

## Supporting information


**Figure S1.** Flow of participants through the study
Table S1 Characteristics of those with probable sarcopenia by whether appendicular lean mass measured or not
Table S2 Characteristics of those with probable sarcopenia by appendicular lean mass status
