# Supplementary material for: Sarcopenia, long‐term conditions, and multimorbidity: findings from UK Biobank participants
Source: J Cachexia Sarcopenia Muscle. 2019 Dec 30;11(1):62–8. doi: 10.1002/jcsm.12503 (PMC7015236; doi:10.1002/jcsm.12503)
Supplement: Supplementary file 1 — Figure S1. Flow of participants through the study Table S1 Characteristics of those with probable sarcopenia by whether appendicular lean mass measured or not Table S2 Characteristics of those with probable sarcopenia by appendicular lean mass status [file JCSM-11-62-s001.docx]

# Supplementary Methods Relationship between appendicular fat-free mass and appendicular lean mass from dual-energy x-ray absorptiometry

Appendicular fat-free mass was measured in the baseline assessment of UK Biobank using a Tanita BC-418MA body composition analyser [24]. This device returns the fat-free mass of each limb but does not partition this into bone and lean mass. Appendicular fat-free mass assessed by dual-energy x-ray absorptiometry (DXA) and the Tanita BC-418MA body composition analyser have previously shown to be tightly correlated in 40 individuals aged six to 64 years [42].

We used data from DXA scans UK Biobank imaging assessment (2015 onwards) to examine the relationship between appendicular lean mass (ALM) and appendicular fat-free mass, in order to produce a conversion equation which could be applied to the bioimpedance data. We calculated appendicular lean mass as appendicular fat-free mass less appendicular bone mineral content (all in kg). We used multiple linear regression to produce a conversion equation, including gender as a covariate.

A total of 4,350 participants (52.5% female) of mean age 55.4 (SD 7.6) years had DXA measures available from the imaging assessment. Mean (SD) appendicular fat-free mass and ALM were 18.5 (2.7) and 17.4 (2.6) kg in women, respectively, and 27.3 (3.9) and 25.7 (3.7) kg in men, respectively. We saw a strong correlation between appendicular fat-free mass and appendicular lean mass in both women and men, with correlation coefficients of 0.999 and as shown in the figure below.

**Figure** Relationship between appendicular fat-free mass and lean mass from DXA

From the regression model for the relationship between appendicular fat-free mass and ALM, taking into account age and gender, we produced the following conversion equation:

ALM (kg) = (0.958 * [Appendicular fat-free mass (kg)]) – (0.166 * *G*) – 0.308

*G* taking value 0 if female and 1 if male.

# Supplementary Figure 1 Flow of participants through the study

# Supplementary Table 1 Characteristics of those with probable sarcopenia by whether appendicular lean mass measured or not

| **Characteristic** | **ALM measured** | | **ALM missing** | |
| --- | --- | --- | --- | --- |
| (N = 26671 and | n = 25901 | | n = 770 | |
| all values n (%) unless shown) | (97.1%) | | (2.9%) | |
| Age (mean (SD)) | 59.9 | (7.2) | 60.1 | (7.3) |
| Age category |  |  |  |  |
| 40-49 | 2,860 | (11) | 88 | (11.4) |
| 50-59 | 7,215 | (27.9) | 207 | (26.9) |
| 60-70 | 15,826 | (61.1) | 475 | (61.7) |
|  |  |  |  |  |
| Female gender | 15,994 | (61.8) | 413 | (53.6) |
|  |  |  |  |  |
| Maximum grip kg (mean (SD)) |  |  |  |  |
| Females | 11.8 | (3) | 10.6 | (3.8) |
| Males | 22.1 | (4.2) | 20.9 | (5.2) |
|  |  |  |  |  |
| BMI kg/m2 (mean (SD)) | 28.1 | (5.4) | 28.8 | (6.8) |
| BMI category |  |  |  |  |
| < 18.5 | 267 | (1) | 13 | (1.7) |
| 18.5 <= BMI < 25 | 7,455 | (28.8) | 212 | (27.5) |
| 25 <= BMI < 30 | 10,309 | (39.8) | 273 | (35.5) |
| BMI >= 30 | 7,870 | (30.4) | 272 | (35.3) |
|  |  |  |  |  |
| Conditions(s) by category |  |  |  |  |
| Cardiovascular | 12,886 | (49.8) | 484 | (62.9) |
| Respiratory / ENT | 5,927 | (22.9) | 195 | (25.3) |
| Gastrointestinal | 5,943 | (22.9) | 176 | (22.9) |
| Renal / urology | 1,643 | (6.3) | 68 | (8.8) |
| Endocrine / diabetes | 4,804 | (18.5) | 181 | (23.5) |
| Neuro / psych. | 5,124 | (19.8) | 227 | (29.5) |
| Musculoskeletal / trauma | 10,793 | (41.7) | 346 | (44.9) |
| Haematology / dermatology | 1,852 | (7.2) | 66 | (8.6) |
| Gynaecology / breast | 1,778 | (6.9) | 46 | (6) |
| Immunological / systemic | 2,499 | (9.6) | 65 | (8.4) |
| Infections | 636 | (2.5) | 18 | (2.3) |
| Eye | 1,753 | (6.8) | 80 | (10.4) |
| History of cancer | 2,814 | (10.9) | 91 | (11.8) |
| Number of categories affected |  |  |  |  |
| 0 | 3,022 | (11.7) | 55 | (7.1) |
| 1 | 6,167 | (23.8) | 140 | (18.2) |
| 2 | 6,617 | (25.5) | 207 | (26.9) |
| 3+ | 10,095 | (39) | 368 | (47.8) |
|  |  |  |  |  |
| Falls in last year (n=26294) |  |  |  |  |
| 0 | 17,238 | (67.5) | 401 | (53.3) |
| 1 | 4,278 | (16.7) | 137 | (18.2) |
| 2+ | 4,025 | (15.8) | 215 | (28.6) |
|  |  |  |  |  |
| Self-reported walk speed (n=26284) | |  |  |  |
| Unable to walk | 352 | (1.4) | 49 | (6.6) |
| Slow pace | 6,637 | (26) | 329 | (44.2) |
| Steady average pace | 13,279 | (52) | 271 | (36.4) |
| Brisk pace | 5,271 | (20.6) | 96 | (12.9) |
|  |  |  |  |  |
|  |  |  |  |  |
| ALM, appendicular lean mass. ENT, ear nose and throat. | | |  |  |

# Supplementary Table 2 Characteristics of those with probable sarcopenia by appendicular lean mass status

| **Characteristic** | **Normal ALM for height** | | **Confirmed sarcopenia** | |
| --- | --- | --- | --- | --- |
| (N = 25901 and | n = 24231 | | n = 1670 | |
| all values n (%) unless shown) | (93.6%) | | (6.4%) | |
| Age (mean (SD)) | 59.8 | (7.2) | 61.0 | (6.7) |
| Age category |  |  |  |  |
| 40-49 | 2,731 | (11.3) | 129 | (7.7) |
| 50-59 | 6,797 | (28.1) | 418 | (25) |
| 60-70 | 14,703 | (60.7) | 1,123 | (67.2) |
|  |  |  |  |  |
| Female gender | 14,952 | (61.7) | 1,042 | (62.4) |
|  |  |  |  |  |
| Maximum grip kg (mean (SD)) |  |  |  |  |
| Females | 11.8 | (2.9) | 11.5 | (3.3) |
| Males | 22.2 | (4.2) | 21.5 | (4.6) |
|  |  |  |  |  |
| BMI kg/m2 (mean (SD)) | 28.6 | (5.2) | 20.7 | (2.1) |
| BMI category |  |  |  |  |
| < 18.5 | 29 | (0.1) | 238 | (14.3) |
| 18.5 <= BMI < 25 | 6,048 | (25) | 1,407 | (84.3) |
| 25 <= BMI < 30 | 10,284 | (42.4) | 25 | (1.5) |
| BMI >= 30 | 7,870 | (32.5) | 0 | (0) |
|  |  |  |  |  |
| Conditions(s) by category |  |  |  |  |
| Cardiovascular | 12,284 | (50.7) | 602 | (36) |
| Respiratory / ENT | 5,516 | (22.8) | 411 | (24.6) |
| Gastrointestinal | 5,579 | (23) | 364 | (21.8) |
| Renal / urology | 1,537 | (6.3) | 106 | (6.3) |
| Endocrine / diabetes | 4,614 | (19) | 190 | (11.4) |
| Neuro / psych. | 4,800 | (19.8) | 324 | (19.4) |
| Musculoskeletal / trauma | 10,143 | (41.9) | 650 | (38.9) |
| Haematology / dermatology | 1,739 | (7.2) | 113 | (6.8) |
| Gynaecology / breast | 1,669 | (6.9) | 109 | (6.5) |
| Immunological / systemic | 2,335 | (9.6) | 164 | (9.8) |
| Infections | 571 | (2.4) | 65 | (3.9) |
| Eye | 1,618 | (6.7) | 135 | (8.1) |
| History of cancer | 2,606 | (10.8) | 208 | (12.5) |
| Number of categories affected |  |  |  |  |
| 0 | 2,801 | (11.6) | 221 | (13.2) |
| 1 | 5,701 | (23.5) | 466 | (27.9) |
| 2 | 6,199 | (25.6) | 418 | (25) |
| 3+ | 9,530 | (39.3) | 565 | (33.8) |
|  |  |  |  |  |
| ALM/height2 kg/m2 (mean (SD)) | |  |  |  |
| Females | 7.3 | (0.9) | 5.8 | (0.2) |
| Males | 8.8 | (1.2) | 6.6 | (0.4) |
|  |  |  |  |  |
| Falls in last year (n=25541) |  |  |  |  |
| 0 | 16,080 | (67.3) | 1,158 | (70) |
| 1 | 4,012 | (16.8) | 266 | (16.1) |
| 2+ | 3,794 | (15.9) | 231 | (14) |
|  |  |  |  |  |
| Self-reported walk speed (n=25539) | |  |  |  |
| Unable to walk | 323 | (1.4) | 29 | (1.8) |
| Slow pace | 6,262 | (26.2) | 375 | (22.7) |
| Steady average pace | 12,492 | (52.3) | 787 | (47.7) |
| Brisk pace | 4,813 | (20.1) | 458 | (27.8) |
|  |  |  |  |  |
|  |  |  |  |  |
| ALM, appendicular lean mass. ENT, ear nose and throat. | | |  |  |

The above table divides those with weak grip strength (at least probable sarcopenia) and ALM measurement into those with normal ALM-for-height, and those with reduced ALM-for-height (confirmed sarcopenia).
